# Intranasal Neuropeptide Y Blunts Lipopolysaccharide-Evoked Sickness Behavior but Not the Immune Response in Mice

**DOI:** 10.1007/s13311-019-00758-9

**Published:** 2019-07-23

**Authors:** Geraldine Zenz, Aitak Farzi, Esther E. Fröhlich, Florian Reichmann, Peter Holzer

**Affiliations:** 1 Research Unit of Translational Neurogastroenterology, Division of Pharmacology, Otto Loewi Research Center, Medical University of Graz, Universitätsplatz 4, A-8010 Graz, Austria; 2 BioTechMed-Graz, Mozartgasse 12, A-8010 Graz, Austria

**Keywords:** Cytokines, Hypothalamic-pituitary-adrenal axis, Immune-brain axis, Lipopolysaccharide, Neuropeptide Y, Sickness behavior

## Abstract

Neuropeptide Y (NPY) has been demonstrated to exert stress buffering effects and promote resilience. Non-invasive intranasal (IN) application of NPY to rodents is able to mitigate traumatic stress-induced behavioral changes as well as dysfunction of the hypothalamic-pituitary-adrenal (HPA) axis. However, it is unknown whether IN NPY could prevent the behavioral, pro-inflammatory and neurochemical responses to peripheral immune activation by the Toll-like receptor 4 (TLR4) stimulant lipopolysaccharide (LPS). Therefore, we analyzed the effects of IN NPY (100 μg) on the behavioral sickness response (reduced locomotion and exploration) and the underlying molecular mechanisms, 3 h and 21 h after intraperitoneal injections of LPS (0.03 mg/kg) in male C57BL/6N mice. The acute behavioral sickness response was significantly dampened by pretreatment with IN NPY 3 h after LPS injection. This effect was accompanied by diminished weight loss and lowered plasma corticosterone (CORT) levels 21 h after LPS injection. In contrast, acute circulating cytokine levels and hypothalamic cytokine mRNA expression remained unaltered by IN NPY, which indicates that the peripheral and cerebral immune response to LPS was left undisturbed. Our findings are in agreement with the reported activity of NPY to dampen the response of the HPA axis to stress. We propose that IN NPY ablates sickness behavior at a site beyond the peripheral and cerebral cytokine response, an action that is associated with reduced activity of the HPA axis as determined by decreased plasma CORT.

These results indicate that IN NPY administration may be relevant to the management of neuropsychiatric disorders arising from immune-induced neuroendocrine dysfunction.

**Electronic supplementary material:**

The online version of this article (10.1007/s13311-019-00758-9) contains supplementary material, which is available to authorized users.

## Introduction

Bacterial infections are associated not only with stimulation of the immune system in the periphery, but also with elevated cytokine expression and changed transmitter release within the brain leading to distinct alterations of behavior. Intraperitoneal (i.p.) administration of lipopolysaccharide (LPS), an outer cell wall component of gram-negative bacteria and Toll-like receptor 4 (TLR4) agonist, has long been known to trigger sickness behavior which includes anorexia and a decrease in locomotion, exploration and social interaction [1]. It is commonly assumed that the effects of i.p. administered LPS replicate the reactions to increased translocation of LPS from the microbiota in the gut lumen under conditions of enhanced intestinal mucosal permeability [2]. Enhanced translocation of LPS has been associated with pathologies such as chronic fatigue syndrome and other neuropsychiatric disorders [2].

Attempts to interfere with the detrimental consequences of peripheral immune stress on the brain have identified neuropeptide Y (NPY) as a neurotransmitter of particular interest. NPY is widely expressed in the peripheral and central nervous system of mammals, acting via at least 4 G protein-coupled receptors termed Y1, Y2, Y4 and Y5 [3]. Knockout of Y2 receptors, for instance, has been found to aggravate short- and long-term behavioral effects of LPS, which suggests that NPY may convey resilience to the adverse effects of immune challenge [4, 5]. However, such an effect of NPY has not yet been directly demonstrated. Nevertheless, NPY is known to promote stress buffering effects that might prevent affective disorders and are associated with reduced activation of the hypothalamic-pituitary-adrenal (HPA) axis, which is also implicated in the response to immune stress [6, 7].

Besides intracerebroventricular injection of NPY [8] or adeno-associated viral vector-induced overexpression of NPY [9], IN administration of NPY is another route that bypasses the blood-brain barrier (BBB) through direct nose-to-brain pathways [10–13].

IN NPY has been shown to reach several brain areas, including the hypothalamus, within 30 min in rodents [12, 14, 15] and to suppress behavioral alterations as well as HPA axis activation in a rodent post-traumatic stress disorder (PTSD) protocol [10, 14].

The objective of the present study was to investigate whether IN pretreatment of male C57BL/6N mice with NPY dampens the immune and behavioral effects of i.p. LPS injection in the acute phase of sickness response. In order to obtain information on the site of action where NPY might interfere with LPS-induced sickness behavior, we analyzed the immune-brain axis at the level of (1) cytokine concentrations in blood plasma, (2) expression of neuroinflammation-related cytokines and tight-junction proteins in the hypothalamus, (3) activity of the HPA axis as reflected by hypothalamic corticotropin-releasing hormone (CRH) expression and plasma corticosterone (CORT) levels, and (4) behavior related to locomotion and exploration.

## Materials and Methods

### Ethics Statement and Experimental Animals

Experiments and the number of animals were approved by an ethical committee at the Federal Ministry of Science, Research and Economy of the Republic of Austria (BMWFW-66.010/0122-WF/V/3b/2017). All procedures were accomplished following the Directive of the European Parliament and of the Council of 22 September 2010 (2010/63/EU). Experiments were designed in such a way that the total number of animals used and suffering were minimized.

Male 8-week-old C57BL/6N mice were purchased from Charles River Laboratories (Sulzfeld, Germany) and habituated for at least one week to the new environment before any intervention was undertaken. Animals were kept in groups of two, standard laboratory chow and tap water were provided ad libitum. The room temperature was set at 22 °C, and a relative air humidity of 50% and a 12 h light/dark cycle were maintained. All housing conditions were tightly controlled.

### Reagents and Dosing

NPY (Phoenix Pharmaceuticals, Karlsruhe, Germany, catalog number 049–03) was stored lyophilized at 4 °C until being dissolved in sterile distilled water (10 μg/μl) right before IN applications of 100 μg per mouse [10].

LPS (catalog number tlrl-3pelps, ultrapure, *E. coli* 0111:B4) was purchased from Invivogen (Toulouse, France) and dissolved in pyrogen-free sterile saline provided by the manufacturer. LPS and pyrogen-free sterile saline (0.9% NaCl) were administered i.p. at the same volume (10 μl/g body weight). In order to evoke a sickness response at a subseptic dose of the immune stimulant, mice were injected with 0.03 mg/kg LPS [16].

### Experimental Protocols

#### Intranasal (IN) Applications and i.p. Injections

**Fig. 1:**
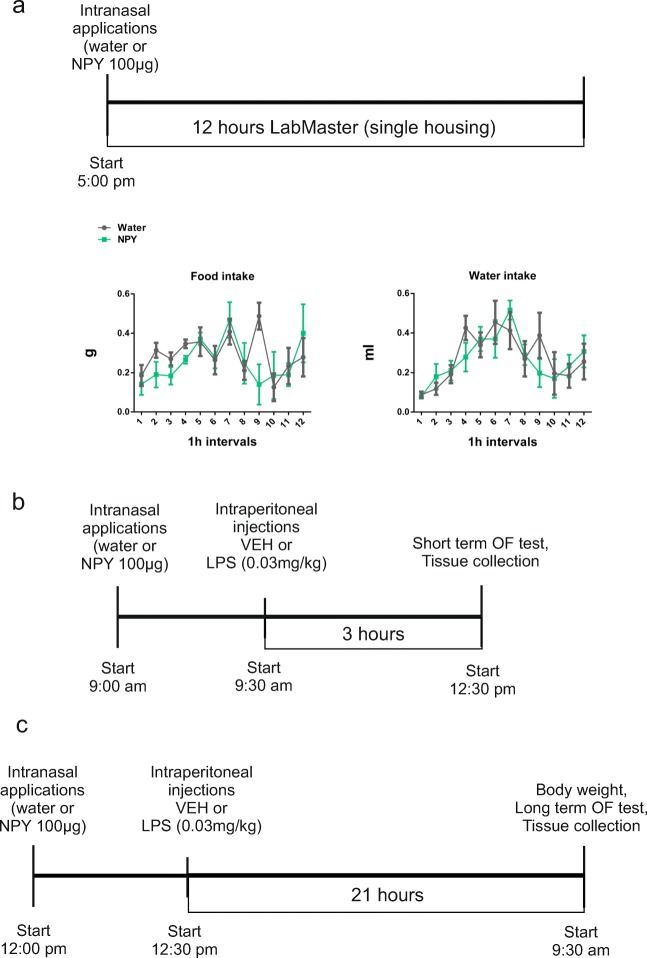
Experimental timeline following IN application of NPY. (a) Ingestive behavior of mice pretreated with NPY (100 μg) or water during the subsequent 12 h. The measurements were taken from mice housed singly in the LabMaster system. Values represent means ± SEM, *n* = 5–7. (b,c) Protocol of readouts taken from mice pretreated with IN water or NPY (100 μg) at the indicated time points, followed by i.p. injections of either vehicle (VEH) or LPS (0.03 mg/kg) exactly 30 min after IN pretreatment. The mice were subjected to the OF test followed by tissue collection either 3 h (b) or 21 h (c) following i.p. treatment

Mice were randomly assigned to one of the experimental groups and, under light isoflurane anesthesia, were IN infused with NPY (100 μg) dissolved in 10 μl water, or water only, as described by others [10]. A pipette was used to slowly apply the fluid in approximately 5 droplets to the nostrils, avoiding contact with the mucosa. Following the application, animals were kept in a tilted backward position for approximately 20 s to facilitate NPY uptake from the nasal cavity to the brain and avoid discharge. Animals were monitored and relocated to their cage only when wakefulness was fully recovered, which usually took place within 5 min. Thirty min after nasal NPY or water application, mice were i.p. injected with either VEH or LPS (Fig. 1).

#### Analysis of Ingestive Behavior Following NPY Treatment

This experiment was conducted to screen for changes in water and food intake of mice treated IN with either water or NPY (Fig. 1a). This method has been described previously [17]. In short, water and food intake of mice (*n* = 5–7) treated IN with either water or NPY was recorded in the homecage-like environment of LabMaster cages (type III; 42.0 × 26.5 × 15.0 cm, length × width × height; TSE Systems, Bad Homburg, Germany). A feeding bin filled with standard rodent chow and a drinking bottle were attached to two weight transducers adherent to the cage lids, which were used to evaluate ingestive behavior throughout the experiment. Recording devices were connected to a personal computer which collected the data with the LabMaster software. The animals were conditioned to the drinking bottles used in the LabMaster system and to single housing for at least 72 h before placing them in the transparent test cages. Another habituation period of at least 24 h enabled the mice to habituate to the LabMaster cages before the experiment was started. Within the LabMaster system, mice were kept one by one in order to enable accurate activity measurements of each mouse. All animals were recorded for 12 h, starting with the IN applications which were performed approximately at 5:00 pm, one h before the dark-cycle began.

#### Open Field (OF) Test

This test was conducted to assess locomotion, exploration and anxiety-like behavior in the novel environment of the OF (Fig. 1b,c), as previously described [17]. Animals (*n* = 8 per group) were habituated to the test room (lights on at 6:00 am, lights off at 6:00 pm, temperature set at 22 °C, relative air humidity set at 50%) for at least 24 h before the behavioral assignment.

**Fig. 2:**
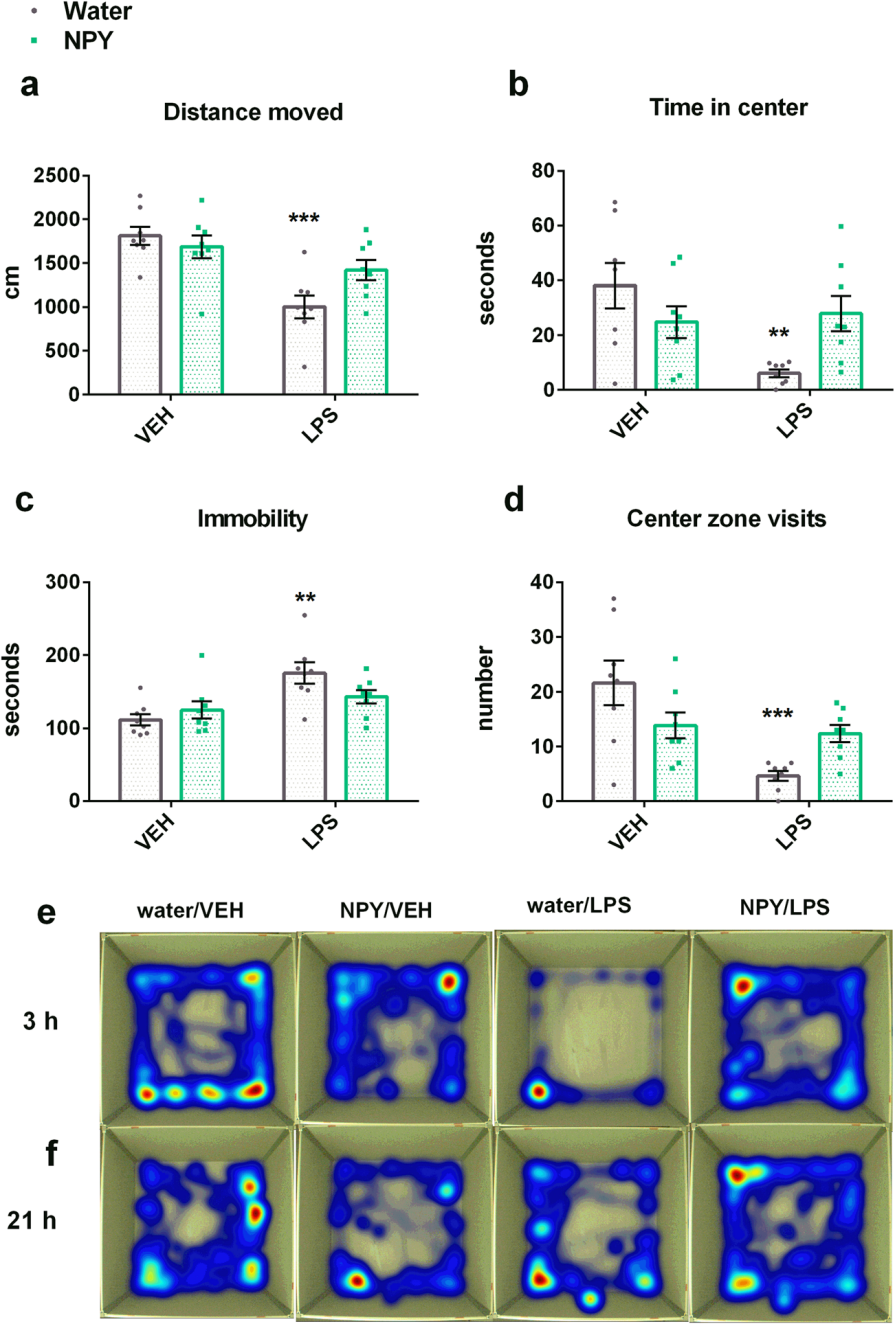
IN pretreatment with NPY prevents i.p. LPS from causing an acute behavioral sickness response. Mice were pretreated with either IN water or NPY (100 μg), followed by i.p. injection of VEH or LPS (0.03 mg/kg) 30 min thereafter. Three h later the open field (OF) test was used to evaluate sickness behavior: (a) distance moved, (b) time spent within the center zone of the OF, (c) time spent immobile, (d) number of central area visits, (e) representative heatmaps of exploratory behavior as recorded 3 h (e) and 21 h (f) after VEH or LPS injection. Values represent means ± SEM, *n* = 8; **p ≤ 0.01, ***p ≤ 0.001 vs. water/VEH (Tukey’s post-hoc test)

The OF, consisting of an opaque gray plastic box (50 × 50 × 30 cm, length × width × height), illuminated by 35 lx at floor level, was divided into a 36 × 36 cm central area and the outer border zone. Mice were placed individually into one corner of the OF and allowed to freely explore the new environment for 5 min. Behavior was recorded and tracked by a video camera above the center of the OF and analyzed with EthoVision XT (Noldus, Wageningen, The Netherlands). This software was also used to generate heatmaps for each mouse, and one representative heatmap for each treatment group at each time point is shown in Fig. 2e,f. The heatmaps visualize the tracks of exploratory behavior of the treatment groups and show the time spent in the different compartments of the OF. After each trial the OF was cleaned with 70% ethanol, followed by water to avoid any olfactory disturbance.

The behavioral assessment was conducted at two time points following i.p. injection of VEH (saline) or LPS (0.03 mg/kg). At the first time point (3 h) the acute sickness response to LPS and at the second time point (21 h) any long-term effects were assessed (Fig. 2, Fig. S1).

### Molecular Readouts

#### Blood and Brain Tissue Harvesting

Directly after the OF test, the animals were deeply anesthetized with i.p. pentobarbital (150 mg/kg). Blood was drawn and brains were harvested to analyze the expression of cytokines and other molecular entities. Blood was drawn with a syringe containing 100 μl of the anticoagulant sodium citrate (3.8%) by cardiac puncture and centrifuged at 1000 × g and 4 °C for 15 min. Supernatant plasma was subsequently frozen on dry ice and stored at −70 °C until used. Brains were harvested after blood was drawn and immediately frozen in 2-methylbutane (Sigma-Aldrich, Vienna, Austria) on dry ice for 10 s. Subsequently, brains were stored until microdissection at −70 °C.

#### Circulating Cytokines

Cytokine levels in the plasma were analyzed with a multiplex immunoassay as described previously [18]. ProcartaPlex™ immunoassays (eBioscience, San Diego, CA, USA) were used to evaluate cytokine concentrations in plasma samples according to the manufacturer’s specifications and as previously described [18]. Cytokines were measured in duplicates, and fluorescent signals were quantified with the Bio-Plex 200 multiplex suspension array system and Luminex® xMAP® technology. Data were analyzed with the Bio-Plex 5.0 software (BioRad, Hercules, CA, USA).

Cytokines that were below detection level were assigned a value of zero as described previously [19, 20]. In the experiments in which cytokine levels were measured 3 h after treatment, all IFN-γ, IL-1β, IL-10, TNF-α, IFN-α and IFN-β samples (*n* = 7–8 per cytokine) and six (out of 7) IL-6 samples in the water/VEH group were below detection limit. In the NPY/VEH group, all IFN-γ, IL-1β, IL-10, TNF-α, IFN-α and IFN-β samples (*n* = 8 per cytokine), six (out of 8) IL-6 samples and one (out of 7) MCP-1 sample were below detection level. In the water/LPS group, all IL-1β and IFN- α samples (n = 7–8 per cytokine) and three (out of 8) IFN-β samples did not exceed the detection limit, which was also the case for all IFN-α samples (n = 7), six (out of 7) IFN-β samples, two (out of 8) IL-1β samples and one (out of 8) IL-10 sample in the NPY/LPS group. When cytokine concentrations were assayed 21 h after vehicle/LPS injection, five (out of 8) MCP-1 samples were below detection limit in the water/VEH group.

#### Brain Microdissection

The hypothalamus was microdissected from the brain in order to assess expression of cytokines and other molecular entities [21]. Microdissection of the hypothalamus (Bregma +0.38 to −2.92) was undertaken on a − 20 °C cold plate (Weinkauf Medizintechnik, Forchheim, Germany) under a stereomicroscope as described previously [21]. Before dissection, the working area and surgical instruments were cleaned with RNase AWAY (Carl Roth, Karlsruhe, Germany). The microdissected hypothalami were collected in MagnaLyser bead tubes (catalog number: 03358 941 001, Roche Diagnostics, Rotkreuz, Switzerland) filled with Precellys beads (Peqlab, Erlangen, Germany) and stored at −70 °C until RNA extraction.

#### RNA Extraction and Nanostring Analysis

mRNA expression patterns in the hypothalamus were measured by Nanostring analysis as described by others [22]. For NanoString hybridization total RNA was isolated from the dissected brain tissue using RNeasy Kit from Qiagen (Hilden, Germany) and quantified with the NanoDrop ND 1000 spectrophotometer (Thermo Fisher Scientific). The NanoString method and analysis was performed as suggested by the supplier’s instructions.

The nCounter PlexSet oligonucleotide and probe design was performed at NanoString Technologies (NanoString Technologies, Seattle, WA, USA) for 24 genes, including four housekeeping (HK) genes (PPIL3, Ywhaz, Tubb5, UBE2D2; supplementary Table S1). Corresponding oligonucleotides were synthesized at Integrated DNA Technologies (Leuven, Belgium). Titration reactions were performed according to supplier’s instructions with 20 ng, 100 ng and 200 ng to determine the required RNA amount for hybridization reaction. 1190 ng total RNA per sample was used for PlexSet hybridization reaction for 20 h according to manufacturer’s instructions.

Samples were processed on a nCounter MAX prep station (NanoString Technologies, Seattle, WA, USA) at the CoreFacility Molecular Biology of the Medical University of Graz. Cartridges were scanned in a generation II nCounter Digital Analyzer (NanoString Technologies, Seattle, WA, USA). RCC files (nCounter data files) were used for data analysis.

RCC files were imported into the NanoStrings nSolver 4.0 analysis software and raw data pre-processing and normalization was performed according to manufacturer’s instructions for standard procedures (positive normalization to geomean of top 3 positive controls, codeset content normalization using housekeeping genes Ppil3, Ywhaz, Tubb5, Ube2d2 and codeset calibration with the reference sample).

Mean mRNA values of the water/VEH control group were used to calculate the fold-change differences between the treatment groups in their expression of each gene. Genes that were significantly altered in their mRNA expression at either time point under study are shown in the main text, all other genes are reported in the supplementary information.

### Statistics and Artwork

Two-way ANOVA was performed to analyze all experiments, followed by Tukey’s post-hoc test for multiple group comparison using GraphPad® Prism5 (GraphPad Software Inc., La Jolla, CA, United States) software. *p*-Values ≤0.05 were regarded significant. Artwork was created using Prism GraphPad® Prism5 and *CorelDRAW*® Graphics Suite 2019 (for Windows).

## Results

### IN NPY Does Not Alter Food or Water Intake

Ingestive behavior did not differ between mice treated with IN NPY or water as measured during the 12 h period following application (Fig. 1a).

### IN NPY Abates Acute Behavioral Sickness in Response to LPS

Exploratory behavior in the novel environment of the OF differed significantly between the experimental groups 3 h following i.p. injection of VEH or LPS (Fig. 2). Two-way ANOVA revealed a significant interaction between the effects of IN NPY and i.p. LPS treatments on the distance traveled within the OF (F(1,28)=5.079, *p* ≤ 0.05) and the time spent within the center zone of the OF (F(1,28)=8.476, *p* ≤ 0.01). Furthermore, we found a significant interaction between the IN NPY and i.p. LPS treatments on the number of entries to the center zone (F(1,28)=9.552, p ≤ 0.01) as well as the time spent immobile (F(1,28)=4.185, p ≤ 0.05). These signs of an acute sickness response had disappeared 21 h after i.p. LPS injection when no longer any significant differences between the experimental groups were observed (Supplementary Fig. S1, Fig. 2f).

Tukey’s multiple comparison test showed that only the water/LPS group differed significantly from the water/VEH group in all measured parameters (Fig. 2a-d). Thus, the distance moved, the time spent in the center of the OF and the number of visits to the center zone were all significantly reduced in the water/LPS group, whereas the time of immobility was significantly prolonged (Fig. 2a-d). In contrast, none of the behavioral readouts taken in the NPY/LPS group differed significantly from those measured in the water/VEH and NPY/VEH groups (Fig. 2a-d). Taken together, IN NPY pretreatment prevented i.p. LPS from eliciting an acute behavioral sickness response as revealed in the OF test and reflected by heatmap illustrations (Fig. 2e,f).

### IN NPY Does Not Blunt the LPS-Induced Rise of Circulating Cytokine Concentrations in the Acute Phase of Sickness

**Fig. 3:**
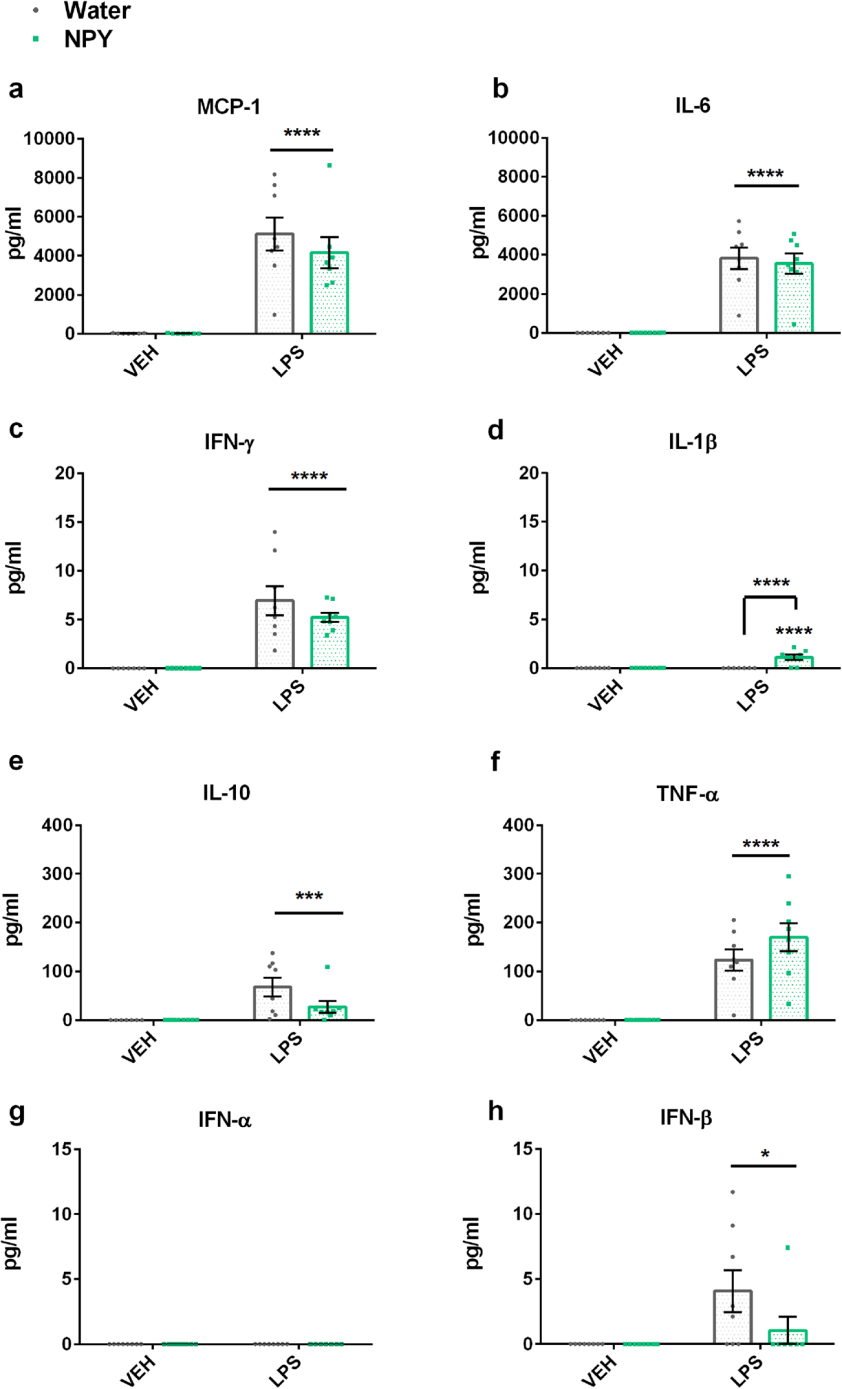
Circulating cytokines are elevated by i.p. LPS injection, in most cases independently of IN pretreatment with NPY. Mice were pretreated with either IN water or NPY (100 μg) and 30 minutes thereafter injected i.p. with VEH or LPS (0.03 mg/kg). Three h later the animals were sacrificed and plasma samples analyzed for circulating cytokine levels (a-h). Values represent means ± SEM, *n* = 7–8; *p ≤ 0.05, ***p ≤ 0.001, ****p ≤ 0.0001 vs. VEH groups (main effect, two-way ANOVA) or as indicated. MCP = monocyte chemoattractant protein, IL = interleukin, IFN = interferon, TNF = tumor necrosis factor

LPS enhanced circulating cytokine levels as measured 3 h following injection (Fig. 3). Two-way ANOVA revealed a strong main effect of i.p. LPS on the release of six cytokines into the blood stream: MCP-1 (F(1,25)=57.78, *p* ≤ 0.0001), IL-6 (F(1,27)=92.28, p ≤ 0.0001), IFN-γ (F(1,27)=55.93, p ≤ 0.0001), IL-10 (F(1,27)=16.46, *p* ≤ 0.001), TNF-α (F(1,28)=66.69, p ≤ 0.0001) and IFN-β (F(1,27)=7.131, *p* ≤ 0.05). There was, however, no interaction between IN NPY pretreatment and i.p. LPS injection, which demonstrates that LPS increased the plasma levels of MCP-1, IL-6, IFN-γ, IL-10, TNF-α and IFN-β independently of the pretreatment with IN NPY. IL-1β showed a different pattern as there was a significant interaction between the effects of IN NPY infusion and i.p. LPS injection on the circulating levels of this cytokine (F(1,27)=16.20, *p* ≤ 0.001). Tukey’s post-hoc test showed that circulating IL-1β was significantly higher in mice treated with NPY/LPS compared to animals treated with water/VEH (*p* < 0.0001) and water/LPS (p < 0.0001) (Fig. 3d).

Twenty one h after i.p. LPS, all measured cytokines except MCP-1 had returned to baseline and were below detection level (data not shown). In contrast, MCP-1 remained elevated in mice treated with water/LPS and NPY/LPS (Supplementary Fig. S2).

### Hypothalamic Expression of Cytokine and Tight-Junction Protein mRNA Is Differentially Modified by IN NPY and i.p. LPS

**Fig. 4:**
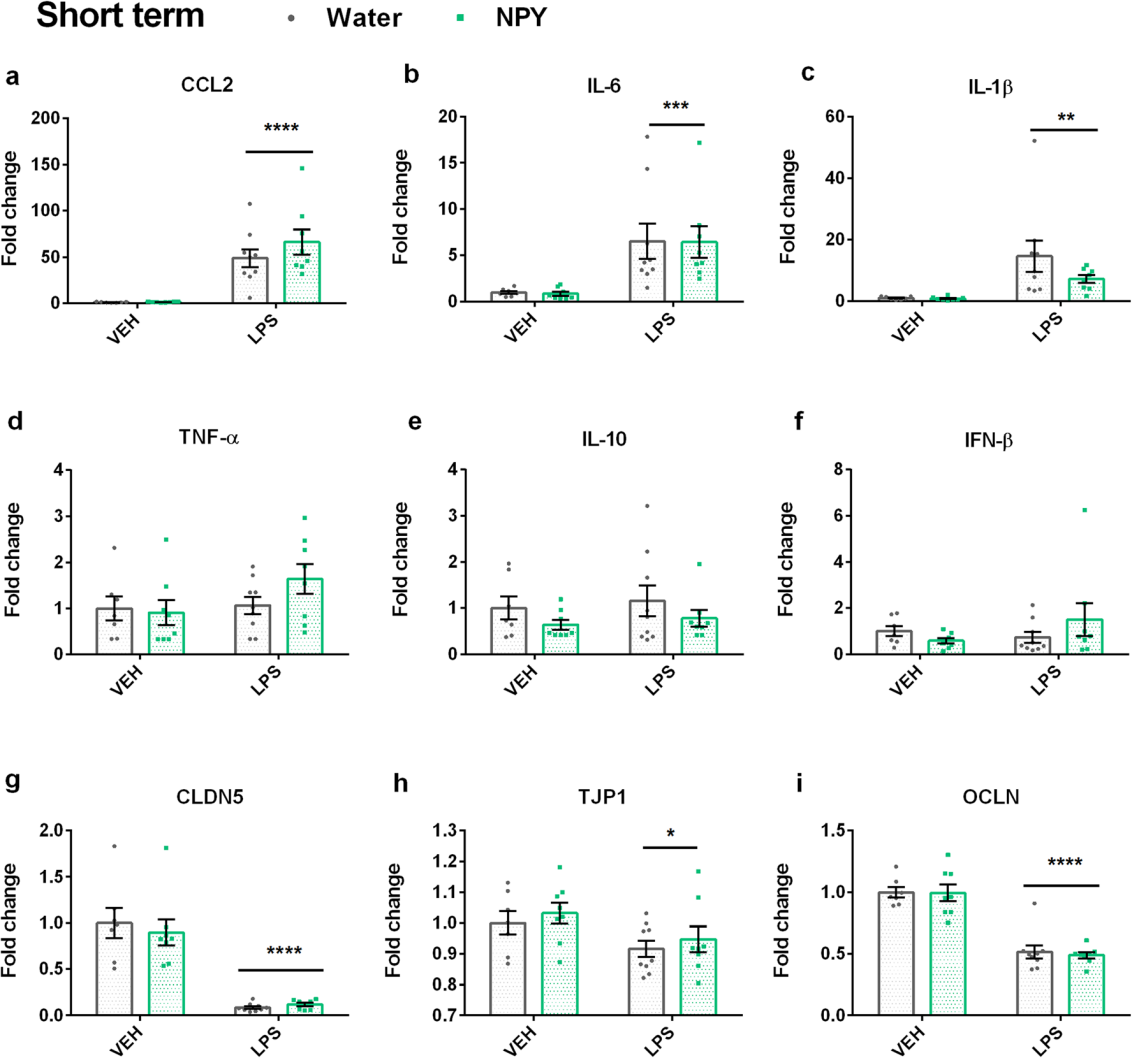
Hypothalamic cytokine and tight junction protein mRNA expression 3 h (short term) after i.p. injection of VEH or LPS. Mice were pretreated with either IN water or NPY (100 μg) and 30 minutes thereafter injected i.p. with VEH or LPS (0.03 mg/kg). Three h later the animals were sacrificed and brain tissue was analyzed for mRNA expression levels of cytokines and tight junction proteins (a-i). Values represent means ± SEM, n = 7–9, *p ≤ 0.05, **p ≤ 0.01, ***p ≤ 0.001, ****p ≤ 0.0001 vs. VEH groups (main effect, two-way ANOVA). CCL = CC-chemokine ligand, IL = interleukin, TNF = tumor necrosis factor, IFN = interferon, CLDN = claudin, TJP = tight-junction protein, OCLN = occludin

**Fig. 5:**
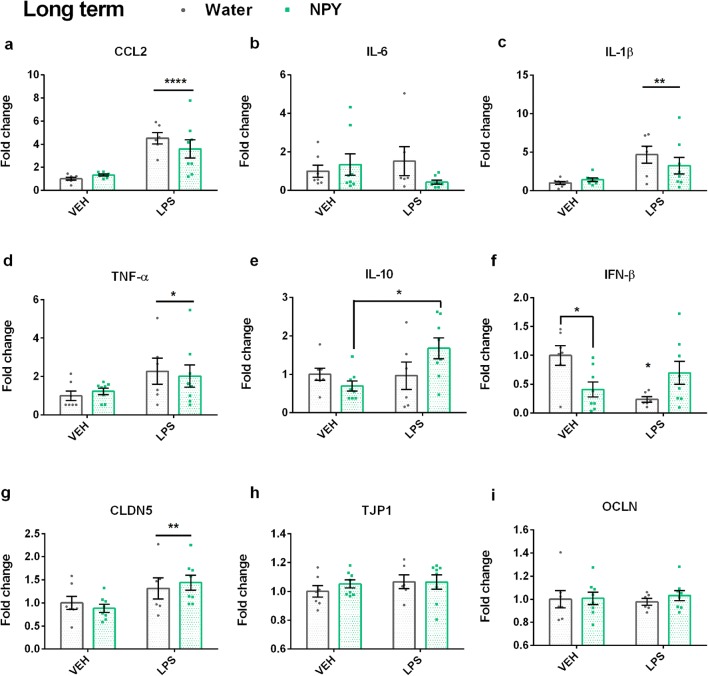
Hypothalamic cytokine and tight junction protein mRNA expression 21 h (long term) after i.p. injection of VEH or LPS. Mice were pretreated with either IN water or NPY (100 μg) and 30 minutes thereafter injected i.p. with VEH or LPS (0.03 mg/kg). Twenty one h later the animals were sacrificed and brain tissue was analyzed for mRNA expression levels of cytokines and tight junction proteins (a-i). Values represent means ± SEM, *n* = 6–8; (a,c,d,g) *p ≤ 0.05, **p ≤ 0.01, ****p ≤ 0.0001 vs. VEH groups (main effect, two-way ANOVA), (e,f) *p ≤ 0.05 as indicated (Tukey’s post-hoc test). CCL = CC-chemokine ligand, IL = interleukin, TNF = tumor necrosis factor, IFN = interferon, CLDN = claudin, TJP = tight-junction protein, OCLN = occludin

Hypothalamic cytokine and tight-junction protein mRNA expression patterns were significantly changed in LPS-treated mice 3 h post-injection, with little to no effects of pretreatment with NPY (Fig. 4). Twenty one h post-treatment, some cytokines were still upregulated in LPS-treated mice (Fig. 5). mRNA expression of genes such as brain-derived neurotrophic factor (BDNF), glutamate receptor GRIN2B, claudin-1 (CLDN1), interferon-α (IFN-α), interferon-γ (IFN-γ) and CRH were not significantly altered by IN NPY pretreatment and i.p. LPS injection 3 h and 21 h post-injection (Supplementary Fig. S3 and S4).

Two-way ANOVA revealed a main effect of i.p. LPS treatment 3 h post-injection (Fig. 4a-c, g-i) for MCP-1, which is referred to as CCL2 in the description of hypothalamic mRNA expression patterns (F(1,28)=41.52, *p* ≤ 0.0001), IL-6 (F(1,28)=16.51, p ≤ 0.001), IL-1β (F(1,28)=11.40, *p* ≤ 0.01), CLDN-5 (F(1,28)=69.15, p ≤ 0.0001), TJP1 (F(1,28)=5.802, *p* ≤ 0.05) and OCLN (F(1,28)=96.25, p ≤ 0.0001). While the expression of cytokines (CCL2, IL-6, IL-1β) was upregulated by LPS, the expression of tight-junction proteins (CLDN-5, TJP1, OCLN) was downregulated (Fig. 4).

Twenty one h following i.p. LPS, CCL2 mRNA and IL-1β mRNA were still significantly upregulated (F(1,25)=34.75, p ≤ 0.0001 and F(1,25)=12.60, *p* ≤ 0.01, respectively) (Fig. 5a, c). CLDN5 mRNA expression also remained changed by LPS but, in contrast to its downregulation 3 h post-LPS (Fig. 4g), was significantly enhanced 21 h post-LPS (F(1,25)=7.783, p ≤ 0.01) (Fig. 5g). A main effect of i.p. LPS was likewise found for TNF-α mRNA expression which was significantly upregulated by LPS (F(1,25)=5.210, *p* ≤ 0.05) 21 h post-LPS (Fig. 5d) but not 3 h post-LPS (Fig. 4d). Furthermore, a significant interaction between the IN NPY and i.p. LPS treatments in the expression of IL-10 (F(1,25)=4.569, p ≤ 0.05) and IFN-β (F(1,25)=11.22, p ≤ 0.01) was found 21 h post-LPS. Post-hoc analysis revealed that IFN-β mRNA expression was significantly decreased (*p* ≤ 0.05) by NPY in the absence of LPS treatment (p ≤ 0.05) (Fig. 5f). On the other hand, NPY pretreatment enhanced (p ≤ 0.05) IL-10 mRNA expression in LPS-injected mice relative to NPY pretreatment of VEH-injected animals (Fig. 5e).

### Hypothalamic Y5 and NR3C1 Gene mRNA Levels Are Changed by i.p. LPS 3 h Post Treatment, Y2 mRNA Expression Is Reduced by NPY/LPS Treatment after 21 h

**Fig. 6:**
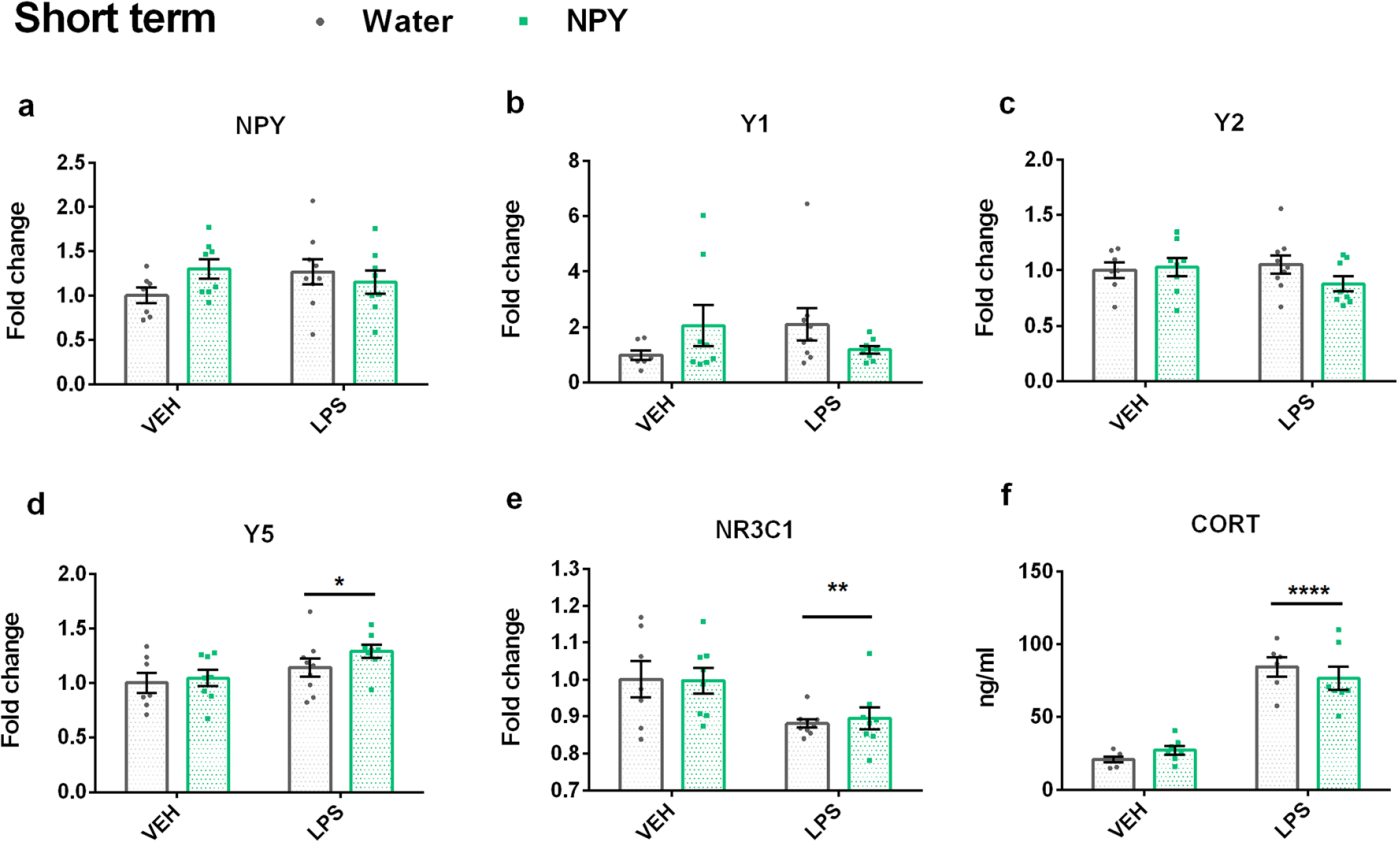
Hypothalamic NPY-related and NR3C1 gene mRNA levels and plasma CORT levels 3 h (short term) after i.p. injection of VEH or LPS. Mice were pretreated with either IN water or NPY (100 μg) and 30 minutes thereafter injected i.p. with VEH or LPS (0.03 mg/kg). Three h later the animals were sacrificed, plasma samples were analyzed for circulating CORT levels and brain tissue was analyzed for mRNA expression levels of NPY-related genes and NR3C1 (a-f). Values represent means ± SEM, (a-e) n = 6–9, (f) n = 6–7, *p ≤ 0.05, **p ≤ 0.01, ****p ≤ 0.0001 vs. VEH groups (main effect, two-way ANOVA). NPY = neuropeptide Y, NR3C1 = nuclear receptor subfamily 3 group C member 1, CORT = corticosterone

**Fig. 7:**
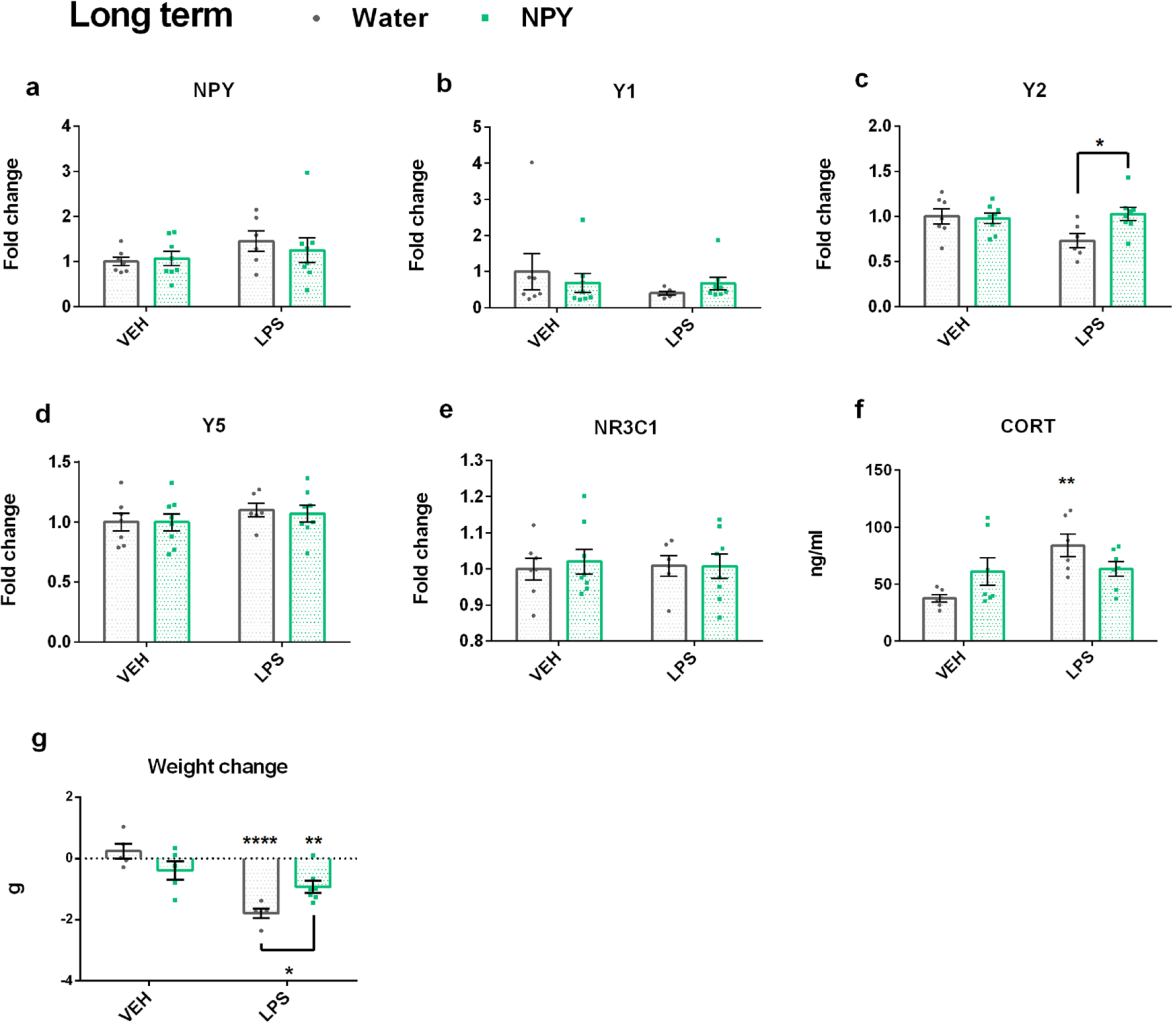
Hypothalamic NPY-related and NR3C1 gene mRNA levels, plasma CORT levels, and weight loss 21 h (long term) after i.p. injection of VEH or LPS. Mice were pretreated with either IN water or NPY (100 μg) and 30 minutes thereafter injected i.p. with VEH or LPS (0.03 mg/kg). Twenty one h later the animals were weighed, thereafter sacrificed, plasma samples were analyzed for circulating CORT levels and brain tissue was analyzed for mRNA expression levels of NPY-related genes and NR3C1 (a-g). Values represent means ± SEM, (a-e) n = 6–9, (f) n = 6–7, (g) n = 5–7; *p ≤ 0.05, **p ≤ 0.01, ****p ≤ 0.0001 vs. water/VEH or as indicated (Tukey’s post-hoc test). NPY = neuropeptide Y, NR3C1 = nuclear receptor subfamily 3 group C member 1, CORT = corticosterone

Patterns of Y5 receptor and NR3C1 receptor mRNA expression were significantly changed 3 h post-injection in LPS-treated mice, with little to no effects of pretreatment with NPY (Fig. 6). Twenty one h post-treatment, a significant interaction between the IN NPY and i.p. LPS treatments was found for the expression of Y2 receptor mRNA (Fig. 7c).

Two-way ANOVA revealed a main effect of i.p. LPS treatment 3 h post-injection for Y5 (F(1,28)=6.014, *p* ≤ 0.05), and NR3C1 (F(1,28)=12.23, *p* ≤ 0.01). While the expression of the Y5 receptor was upregulated by LPS, the expression of the glucocorticoid receptor NR3C1 was downregulated (Fig. 6d,e).

Twenty one h following i.p. LPS, a significant interaction between the IN NPY and i.p. LPS treatments in the expression of Y2 (F(1,25)=4.781, p ≤ 0.05) mRNA was found. Post-hoc analysis revealed that the expression of Y2 receptor mRNA was significantly lower in LPS-injected mice pretreated with water compared to LPS-injected mice pretreated with NPY (p ≤ 0.05) (Fig. 7c).

### LPS-Induced Rise of Circulating CORT Is Blunted by IN Pretreatment with NPY 21 h after Injections

Three h after i.p. injection there was a significant main effect of LPS (F(1,22)=99.64, *p* ≤ 0.0001) on plasma CORT levels which were significantly higher in both groups treated with LPS. Thus, IN NPY did not alter the effect of i.p. LPS to enhance plasma CORT during the acute phase of the behavioral sickness response (Fig. 6f). In contrast, 21 h after i.p. LPS injection we noticed a significant interaction between IN NPY pretreatment and i.p. LPS injection (F(1,22)=6.244, p ≤ 0.05). Tukey’s post-hoc analysis disclosed a significant difference only between the water/LPS and water/VEH group (p ≤ 0.01, Fig. 7f). Unlike mice treated with water/LPS, animals treated with NPY/LPS did not present with elevated CORT levels at that time point.

### LPS-Induced Weight Loss Is Significantly Mitigated by Preceding IN NPY Infusion

An interaction between the effects of IN NPY pretreatment and i.p. LPS injection on weight loss 21 h after i.p. LPS treatment was found by two-way ANOVA (F(1,18)=10.66, p ≤ 0.01). Post-hoc Tukey’s multiple comparison test showed that animals treated with water/LPS or NPY/LPS lost weight when compared to water/VEH-treated animals (p ≤ 0.0001 and p ≤ 0.01, respectively). However, the NPY/LPS group lost significantly less weight than the water/LPS group (p ≤ 0.05, Fig. 7g).

## Discussion

The current data provide direct evidence that IN administration of NPY can interrupt the deleterious manifestations of peripheral immune stimulation on the sickness and neuroendocrine response. This is reflected by the dampened acute behavioral response to peripheral immune stress by LPS, earlier regression of plasma CORT levels and diminished weight loss in mice pretreated with IN NPY. Our findings are in overall agreement with the reported activity of NPY to dampen the response of the HPA axis to stress as reflected by inhibition of CRH neurons in the paraventricular nucleus of the hypothalamus and reduced release of adrenocorticotropic hormone (ACTH) and CORT in rodents [10, 23, 24] and humans [6].

Stimulation of the innate immune system has long been known to induce an acute syndrome of behavioral changes collectively termed sickness response, which may be followed by an increase in anxiety- and depression-like behavior [1, 25]. The TLR4 agonist LPS, a component of the cell wall of gram-negative bacteria, has been reported to stimulate HPA axis activity at a dose of 0.03 mg/kg [16] and to trigger a sickness response (decrease in locomotion, exploration and ingestion) at 0.1 mg/kg [19]. The present study shows that 0.03 mg/kg LPS is in fact able to enhance the formation of pro-inflammatory cytokines in the periphery and brain, to cause neurochemical changes in the brain, to stimulate HPA axis activity and to trigger sickness for a transient period of time. This high potency of LPS is consistent with the concept that translocation of LPS from the gut lumen to the circulation under conditions of enhanced intestinal permeability can impact on brain function and behavior [2].

Visceral inflammation evokes stress responses which can partly be buffered by the resilience-promoting effects of NPY [2]. This entails one of the most important stress response systems mentioned above: the activation of the HPA axis, which is stimulated by immune and other stressors, but is attenuated by NPY [6, 26]. Furthermore, NPY is implicated in the resilience to stressful or traumatic experiences, whereas a dysfunction of the HPA axis and lower circulating NPY levels can be observed in individuals that do not recover from stress easily [7]. Thus, PTSD patients present with lower plasma and cerebrospinal fluid levels of NPY when compared to healthy individuals [27, 28]. The observation that Y2 and Y4 receptor knockout aggravates short- and long-term disturbances of emotional-affective behavior evoked by i.p. LPS has strengthened the hypothesis that the cerebral NPY system is a resilience factor to control the adverse effects of immune challenge in the brain [4, 5, 29]. Although a protective effect of NPY against LPS-evoked sickness has not yet been directly demonstrated, there are in vitro findings that provide circumstantial support for such a role of NPY. Thus, NPY is able to inhibit LPS-evoked IL-1β release and IL-1β-induced phagocytosis and motility of microglial cells in culture [30, 31]. The prime aim of our study was therefore to explore whether NPY acting in the brain would protect against the immune-stimulant and behavioral effects of i.p. LPS injection in the acute phase of sickness behavior.

To address this aim we administered NPY by IN infusion, which represents a non-invasive route of drug delivery to the brain that bypasses the BBB through olfactory and trigeminal nose-to-brain pathways [12, 13, 32, 33]. A comparison of intravenous and IN administration schemes has shown that comparatively large biologically active peptides can be rapidly delivered to the brain following IN administration although, unlike intracerebroventricular injection, IN infusions do not selectively target specific brain regions [33]. Using the IN route of administration, Esther Sabban’s group has already demonstrated that IN NPY ameliorates molecular and behavioral alterations in a rodent model of PTSD [10, 11, 14] and others have shown that IN NPY suppresses microglia activation and cerebral TNF-α expression in a transgenic murine model of Huntington’s disease [15].

Since IN infusion of NPY at several dosages has been shown to reach distinct rodent brain areas including the hypothalamus within 30 min after its infusion [12, 14, 15], we administered NPY IN at a frequently used dose (100 μg) 30 min before i.p. injection of LPS. At the low dose of 0.03 mg/kg, this TLR4 stimulant was able to evoke a transient sickness response as revealed by a reduction of locomotion and exploration 3 h after its injection to mice pretreated with water (the vehicle for NPY). In NPY-pretreated mice, however, LPS failed to evoke signs of sickness behavior in the OF test, which indicates that IN NPY protected from the behavioral disturbances induced by LPS. In this effect NPY was very potent because, in the dosage used, the peptide was devoid of an effect on ingestive behavior despite its reported activity to stimulate food intake [34]. The ability of IN NPY to prevent LPS-evoked sickness raised the question as to the site of action where NPY interferes with the effect of peripheral immune challenge on brain function and behavior. Given that i.p. LPS is known to stimulate the formation of pro-inflammatory cytokines in the periphery, to modify the BBB, to induce neuroinflammatory processes in the central nervous system, to activate the HPA axis and to alter neuronal signaling in the brain [1, 2, 25, 35], the second aim of our study was to dissect the impact of IN NPY on these distinct levels of the immune-brain axis.

As reported previously [1, 18, 19], LPS enhanced circulating cytokine levels in a pattern typical of TLR4-mediated immune stimulation. The LPS-induced rise of circulating cytokines seen 3 h post-injection was transient because 21 h post-LPS the plasma levels of the measured cytokines, except MCP-1, were no longer elevated. The ability of LPS to stimulate peripheral cytokine release remained unabated by IN NPY, which indicates that IN NPY does not interfere with the effect of LPS on the peripheral immune system. This finding is consistent with the observation that the concentration of NPY in the rodent plasma, unlike that in the cerebrospinal fluid, does not increase 30 min after IN administration of the peptide [11]. In view of these circumstances it was unexpected to note that circulating IL-1β in LPS-treated mice was enhanced by IN NPY. This finding is reminiscent of NPY’s pro-inflammatory activity outside the central nervous system [29] as exemplified by NPY-induced secretion of IL-1β from peritoneal macrophages and human whole blood cells [36, 37]. LPS alone failed to enhance circulating IL-1β, which might be due to the relatively low dose or ultrapure quality of LPS used in this study. The latter explanation is supported by the finding that ultrapure LPS obtained from the same supplier fails to elevate circulating IL-1β levels even when administered at a higher dose [18]. In view of these observations it appears unlikely that circulating IL-1β contributes to the neuroendocrine and behavioral effects of LPS in the current experimental setting.

Peripheral LPS has long been known to activate cerebral microglia and induce the expression of pro-inflammatory cytokines in the brain [1, 2, 38–40]. Like the LPS-induced rise of plasma cytokines, the LPS-induced expression of cytokine mRNA in the hypothalamus remained unabated by IN NPY. We focused on this brain region because LPS is known to enhance neuronal activity in this area as shown by c-Fos expression [16, 19, 41] and because the hypothalamus is a central interface in the cerebral response to immune challenge, stress and other adverse conditions impacting on mental health [42, 43]. It should not go unnoticed that the effect of LPS to enhance the expression of cytokine mRNA in the hypothalamus was more prolonged than its effect to raise plasma cytokines. Thus, the hypothalamic upregulation of several cytokines was still evident 21 h after immune stimulation, which is in line with a previous study measuring cytokine mRNA levels 26 h post-LPS [19]. This observation also speaks against the contention that the hypothalamic cytokine expression may, at least in part, reflect the influx of peripheral cytokine-producing immune cells during the neuroinflammatory process [44]. This argument is further substantiated by the different cytokine profile observed in the plasma and hypothalamus. For instance, 3 h after i.p. LPS injection, IL-1β was upregulated only in the brain, whereas TNF-α, INF-β, INF-γ and IL-10 were upregulated only in the periphery.

The observation that IL-1β expression was enhanced by i.p. LPS in the brain but not in the periphery is worth considering with regard to the fact that the current study was conducted with male mice. I.p. administration of LPS to mice has been reported to enhance the peripheral and cerebral expression of cytokines and to elicit behavioral reactions in a sex-dependent manner [45, 46]. Specifically, the enhancement of the circulating levels of IL-1β is more pronounced in adult female than in male mice, whereas the expression of IL-1β in the prefrontal cortex is more pronounced in male than in female mice [46]. It is also noteworthy that NPY pretreatment caused a delayed increase in hypothalamic IL-10 mRNA expression in LPS-injected mice. Since IL-10 is an anti-inflammatory cytokine [47, 48], this finding may reflect an autoregulatory role of the cytokine and is consistent with the anti-inflammatory activity profile of NPY in the central nervous system [27, 29]. This neuroprotective profile of the peptide also manifests itself in the delayed downregulation of IFN-β mRNA expression caused by NPY in the absence of LPS treatment.

Signaling from the peripheral immune system to the brain is controlled by the BBB and its regulatory mechanisms [1, 2, 49, 50]. Neuroinflammatory processes within the brain associated with upregulation of pro-inflammatory cytokines including CCL2 have been shown to disrupt BBB integrity [51–53]. For this reason, we investigated the hypothalamic expression of tight junction proteins CLDN5, TJP1 and OCLN, which are important factors regulating paracellular permeability of the BBB [54]. The expression of all three tight-junction proteins was significantly downregulated in LPS-treated mice 3 h post-injection. Whether these molecular changes translate to a functional deficit of the BBB awaits to be examined because the BBB has been found to be relatively resistant to LPS-induced disruption [44]. The effect of LPS on tight junction protein expression remained unaltered by IN NPY, which indicates that the peptide did not interfere with the adverse effect of peripheral immune challenge and cerebral neuroinflammation on the molecular integrity of the BBB. It is worth noting that the downregulation of CLDN5 mRNA seen 3 h post-LPS was converted to an upregulation of the tight-junction protein 21 h post-LPS. This observation may reflect a compensatory process aimed at restoration of molecular BBB integrity.

Stimulation of murine PRRs such as TLR4 by LPS has previously been found to activate the HPA axis and cause the release of CORT [4, 18, 19, 55]. The current study shows that IN NPY prevented the LPS-induced rise of plasma CORT 21 h after i.p. LPS injection but failed to blunt the CORT surge during the acute phase of sickness behavior 3 h post-LPS. This effect of NPY was paralleled by its ability to ameliorate the late body weight loss seen 21 h post-LPS, another manifestation of the sickness response to LPS. Given that basal food and water intake was not changed by IN NPY, it is likely that the NPY-induced alleviation of LPS-induced weight loss is due to a regulatory influence on metabolism and energy homeostasis [56]. Such a beneficial effect of NPY is in keeping with a report that TLR4-mediated weight loss due to Bacille Calmette–Guérin infection is aggravated by NPY knockout [17]. In addition, central administration of NPY has been found to counteract the anxiogenic effect of CRH [57, 58] and the ACTH-induced impairment of stress coping [57, 58]. These reports support the conclusion drawn from the current study that NPY enforces resilience to stress caused by immune activation.

**Fig. 8:**
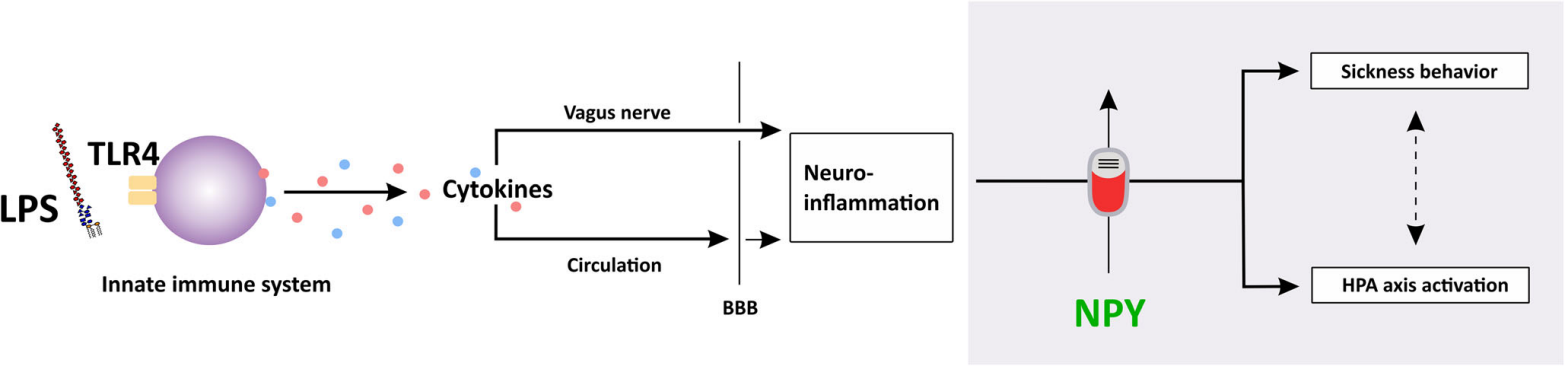
Scheme depicting the presumed site of action at which NPY interferes with the neuroendocrine and behavioral effects of i.p. LPS. BBB = blood-brain barrier, HPA = hypothalamus-pituitary-adrenal, NPY = neuropeptide Y, TLR4 = Toll-like receptor 4, LPS = lipopolysaccharide

While the immune challenge used in the current study was too weak to significantly modify CRH mRNA expression at the investigated time points in the hypothalamus, it resulted in a downregulation of hypothalamic glucocorticoid receptor NR3C1 mRNA 3 h post-LPS when the increase in plasma CORT was largest. Although this observation requires further analysis, it is consistent with a CORT-mediated negative feedback on HPA axis activity [59]. Figure 8 provides a graphical scheme of the presumed site of action at which IN NPY is likely to interfere with the neuroendocrine and behavioral effects of i.p. LPS.

Given the role NPY plays in LPS- and stress-induced alterations of brain function and behavior [3, 29, 60] we examined the expression of NPY and three of its receptors (Y1, Y2, Y5) in the hypothalamus of LPS-treated mice following NPY pretreatment. During the acute phase of LPS-induced sickness, only Y5 receptor mRNA was increased in the hypothalamus irrespectively of NPY pretreatment, an effect that was no longer visible 21 h post-LPS. At this time point, however, Y2 receptor mRNA expression was significantly higher in LPS-injected mice pretreated with NPY relative to LPS-injected mice without NPY treatment. This observation is in keeping with a report that Y2 receptor mRNA expression in the rat amygdala is lowered by stress, an effect that is prevented by IN NPY [61]. Furthermore, cultured endothelial cells have been shown to reduce Y2 receptor, and increase Y5 receptor expression when treated with cytokines [62]. In the current study, the reduction of Y2 expression was blunted by IN NPY, reflected by lower Y2 expression levels in water/LPS-treated mice compared to NPY/LPS-treated animals. Although the changes in Y2 and Y5 receptor mRNA expression hint at distinct alterations of the cerebral NPY system following peripheral immune activation, they require a thorough analysis of the LPS dose-effect and time-effect relationship before firm conclusions can be drawn. Since deletion of Y2 as well as Y4 receptors impairs the resilience to LPS-induced sickness [4, 5], the functional implications of Y2, Y4 and other Y receptors need ultimately be probed by selective receptor antagonists.

In conclusion, this is the first study to show that IN pretreatment with NPY prevents the behavioral sickness response to peripheral immune stimulation by LPS-induced TLR4 activation. In analyzing the site of this action we found that IN NPY fails to counteract the effect of LPS to raise circulating cytokines, downregulate BBB-associated tight junction proteins and stimulate cytokine expression in the hypothalamus (Fig. 8). We therefore conclude that the beneficial effect of IN NPY on sickness behavior takes place in the brain at a site that converts LPS-induced neuroinflammation into behavioral disturbances (Fig. 8). The amelioration of sickness, including the alleviation of weight loss and the rescue of locomotor and exploratory activity, is associated with inhibition of HPA axis activity. In a translational perspective, IN administration of NPY holds promise in the therapeutic control of neuropsychiatric disorders associated with peripheral immune challenge and neuroendocrine disturbances.

## Electronic supplementary material

ESM 1 (PNG 94 kb)

High Resolution Image (TIF 90 kb)

ESM 2 (PNG 26 kb)

High Resolution Image (TIF 22 kb)

ESM 3 (PNG 107 kb)

High Resolution Image (TIF 104 kb)

ESM 4 (PNG 357 kb)

High Resolution Image (TIF 287 kb)

ESM 5 (PDF 459 kb)

ESM 6 (DOCX 28.0 kb)
