# Supplementary figures and images for: Intranasal Neuropeptide Y Blunts Lipopolysaccharide-Evoked Sickness Behavior but Not the Immune Response in Mice
Source: Neurotherapeutics. 2019 Jul 23;16(4):1335–49. doi: 10.1007/s13311-019-00758-9 (PMC6985076; doi:10.1007/s13311-019-00758-9)

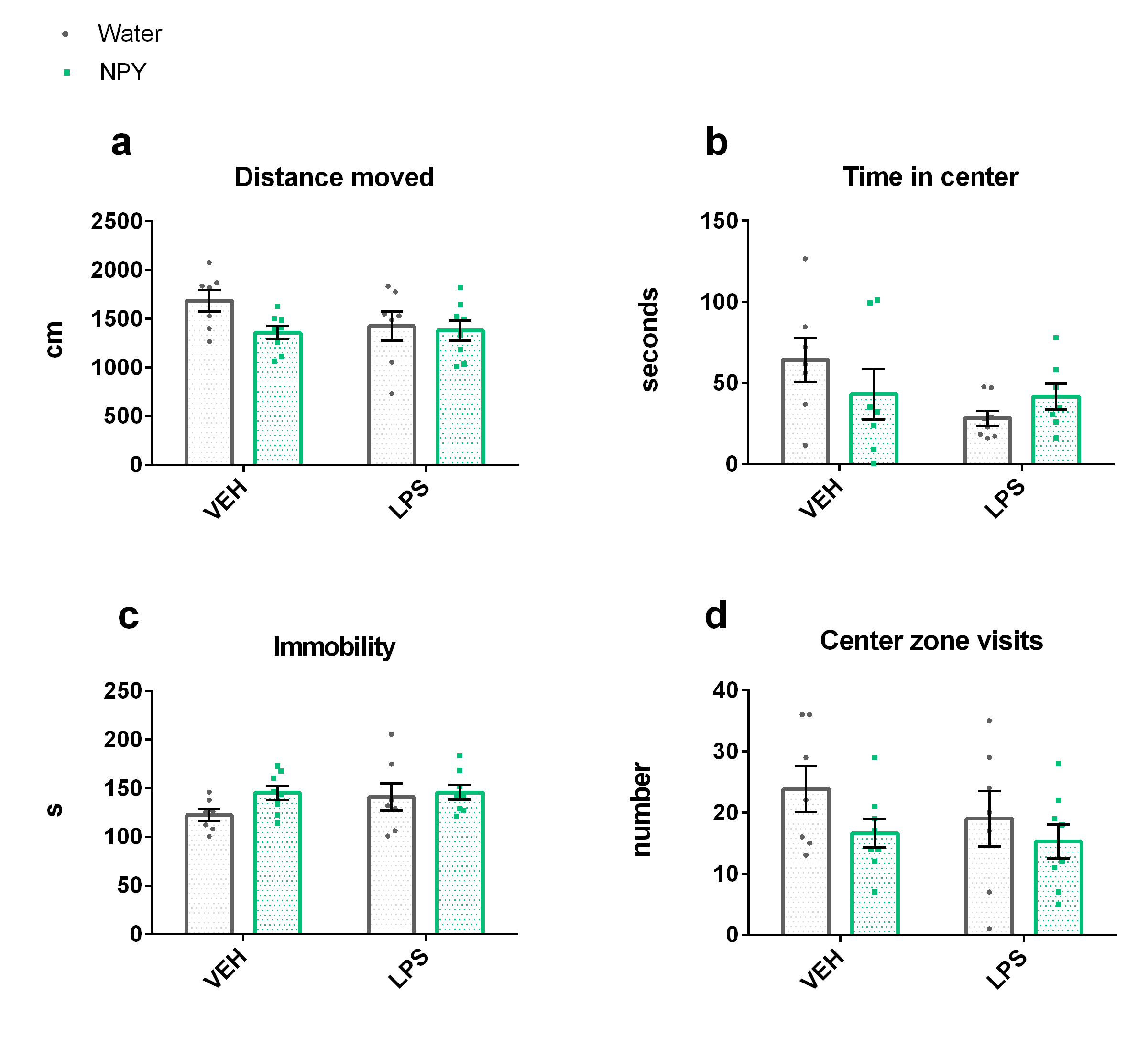

Supplement: Supplementary file 1 — (PNG 94 kb) [file 13311_2019_758_Fig9_ESM.png]

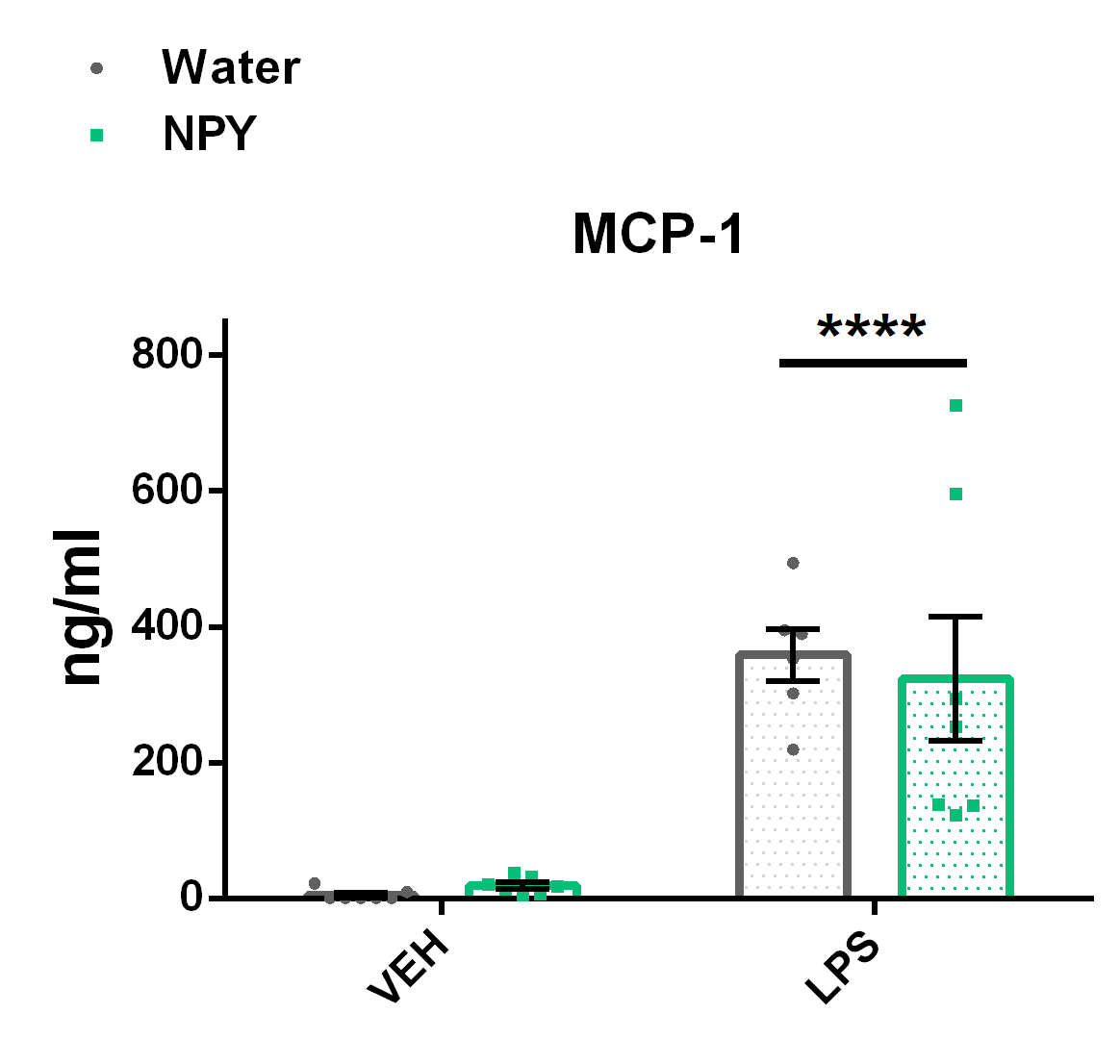

Supplement: Supplementary file 3 — (PNG 26 kb) [file 13311_2019_758_Fig10_ESM.png]

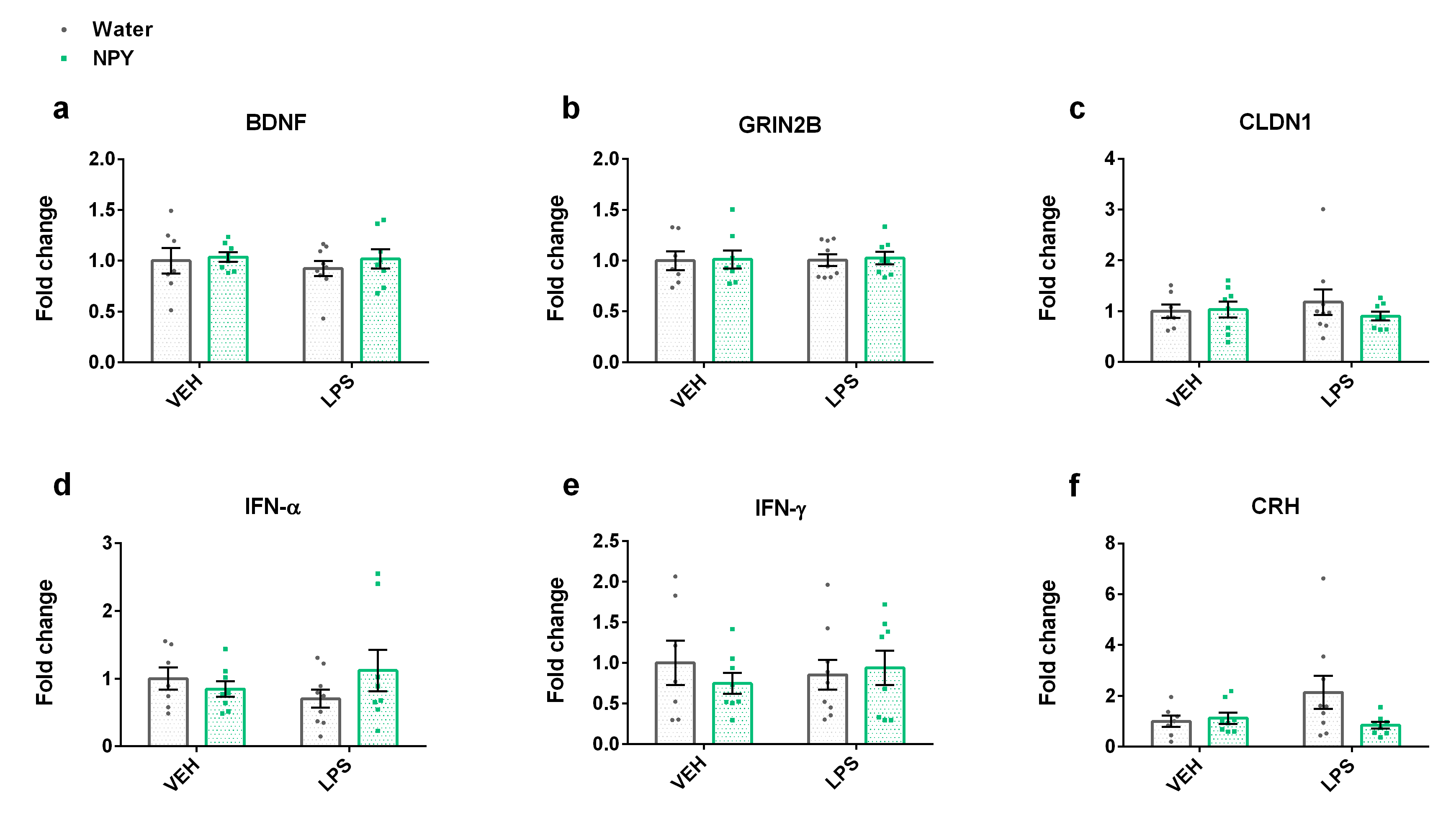

Supplement: Supplementary file 5 — (PNG 107 kb) [file 13311_2019_758_Fig11_ESM.png]

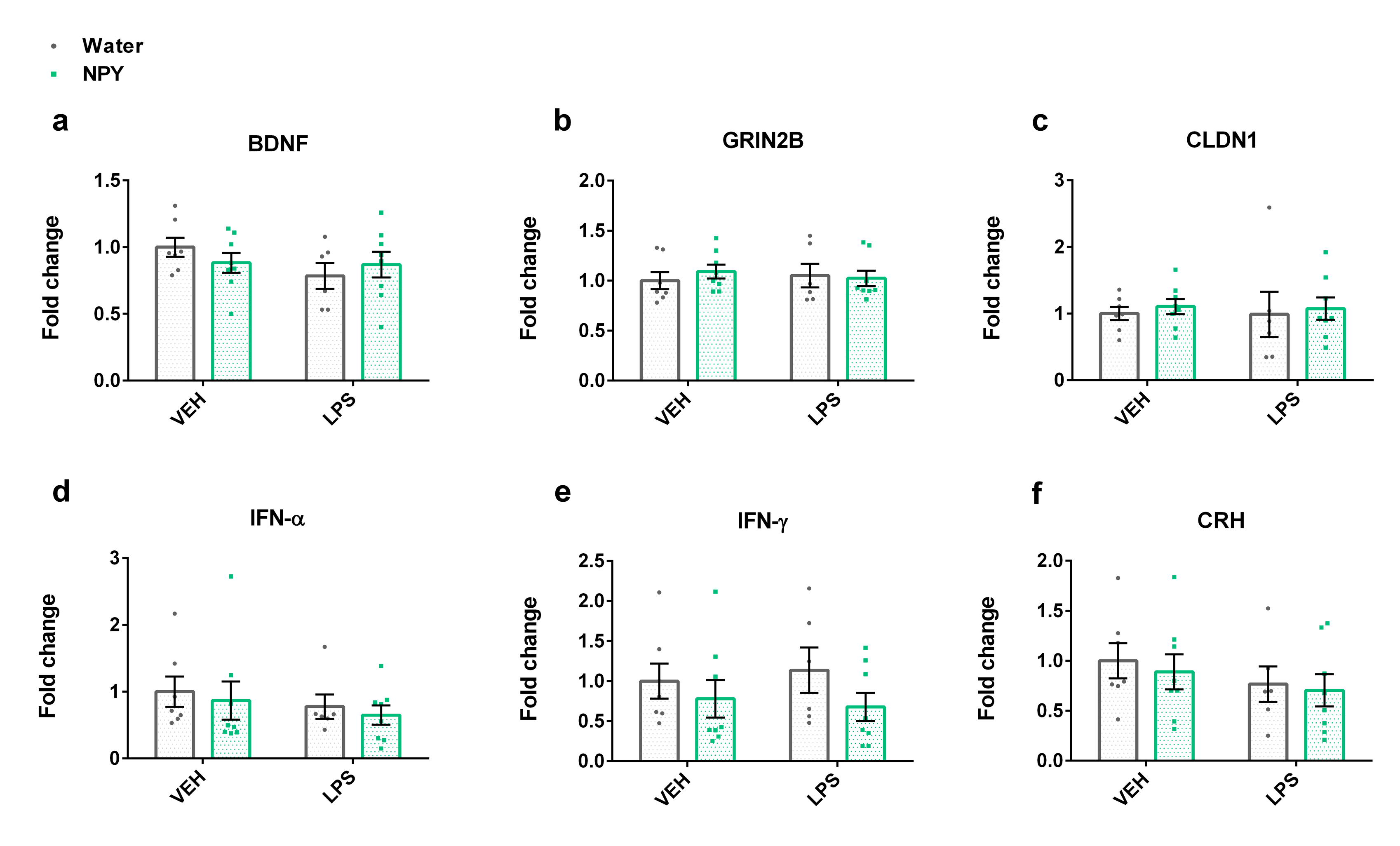

Supplement: Supplementary file 7 — (PNG 357 kb) [file 13311_2019_758_Fig12_ESM.png]
