# Supplementary material for: Intranasal Neuropeptide Y Blunts Lipopolysaccharide-Evoked Sickness Behavior but Not the Immune Response in Mice
Source: Neurotherapeutics. 2019 Jul 23;16(4):1335–49. doi: 10.1007/s13311-019-00758-9 (PMC6985076; doi:10.1007/s13311-019-00758-9)
Supplement: Supplementary file 10 — (DOCX 28.0 kb) [file 13311_2019_758_MOESM6_ESM.docx]

# Supplementary Information

Figure S1 Behavior in the open field 21 h after LPS injection. Mice were pretreated with either IN water or NPY (100 μg) and 30 minutes thereafter injected i.p. with VEH (saline) or LPS (0.03 mg/kg). Twenty one h after i.p. injection the animals were subjected to the open field (OF) test to evaluate the distance moved (a), time spent within the center zone of the OF (b), time spent immobile (c) and the number of central area visits (d). Values represent means +/- SEM, n=7-8.

Figure S2 Plasma MCP-1 (monocyte chemoattractant protein 1) levels 21 h after LPS injection. Mice were pretreated with either IN water or NPY (100 μg) and 30 minutes thereafter injected i.p. with VEH (saline) or LPS (0.03 mg/kg). Twenty one h after i.p. injection the animals were sacrificed and plasma samples analyzed for circulating cytokine levels. Values represent means +/- SEM, n=7-8. ****p≤0.0001 vs. VEH-treated groups (main effect, two-way ANOVA).

Figure S3 Hypothalamic mRNA expression patterns measured 3 h after LPS injection. Mice were pretreated with either IN water or NPY (100 μg) and 30 minutes thereafter injected i.p. with VEH or LPS (0.03 mg/kg). Values represent means +/- SEM, n=6-8. BDNF=brain-derived neurotrophic factor, GRIN2B= glutamate [NMDA] receptor subunit epsilon-2, CLDN1=claudin-1, IFN-α= interferon α, IFN-γ=interferon γ, CRH= corticotropin-releasing hormone.

Figure S4 Hypothalamic mRNA expression patterns measured 21 h after LPS injection. Mice were pretreated with either IN water or NPY (100 μg) and 30 minutes thereafter injected i.p. with VEH or LPS (0.03 mg/kg). Values represent means +/- SEM, n=7-8. BDNF=Brain-derived neurotrophic factor, GRIN2B= glutamate [NMDA] receptor subunit epsilon-2, CLDN1=claudin-1, IFN-α= interferon α, IFN-γ=interferon γ, CRH= corticotropin-releasing hormone.

Table 1 PlexSet Design Genes and Target Sequences used for the PlexSet Design.

| nCounter PlexSet Design Details | | | | | | | | | | | | | |
| --- | --- | --- | --- | --- | --- | --- | --- | --- | --- | --- | --- | --- | --- |
| Report Date: 17-Apr-18 | |  |  |  |  |  |  |  |  | |  |  | |
| **Customer Name** | **Accession** | **Position** | **Target Sequence** | **A Tm** | **B Tm** | **Flags** | **HUGO Gene** | **Species** | | **NSID** | **Tag** | | **Design Remarks** |
| IFN-g (Interferon gamma) | NM_008337.1 | 96-195 | CTAGCTCTGAGACAATGAACGCTACACACTGCATCTTGGCTTTGCAGCTCTTCCTCATGGCTGTTTCTGGCTGTTACTGCCACGGCACAGTCATTGAAAG | 82 | 79 |  | Ifng | Mus musculus | | NM_008337.1:95 | T001 | |  |
| IFN-a (Interferon alpha) | NM_010502.2 | 355-454 | CTGCAAGGCTGTCTGATGCAGCAGGTGGGGGTGCAGGAATTTCCCCTGACCCAGGAAGATGCCCTGCTGGCTGTGAGGAAATACTTCCACAGGATCACTG | 88 | 87 | X | Ifna1 | Mus musculus | | NM_010502.2:354 | T002 | | also targets several other interferon alpha genes @ >90% |
| IFN-b (Interferon beta) | NM_010510.1 | 336-435 | GATGAACTCCACCAGCAGACAGTGTTTCTGAAGACAGTACTAGAGGAAAAGCAAGAGGAAAGATTGACGTGGGAGATGTCCTCAACTGCTCTCCACTTGA | 79 | 82 |  | Ifnb1 | Mus musculus | | NM_010510.1:335 | T003 | |  |
| IL-6 (Interleukin 6) | NM_031168.1 | 41-140 | CTCTCTGCAAGAGACTTCCATCCAGTTGCCTTCTTGGGACTGATGCTGGTGACAACCACGGCCTTCCCTACTTCACAAGTCCGGAGAGGAGACTTCACAG | 82 | 82 |  | Il6 | Mus musculus | | NM_031168.1:40 | T004 | |  |
| IL-1b (Interleukin 1 beta) | NM_008361.3 | 109-208 | TTGACAGTGATGAGAATGACCTGTTCTTTGAAGTTGACGGACCCCAAAAGATGAAGGGCTGCTTCCAAACCTTTGACCTGGGCTGTCCTGATGAGAGCAT | 81 | 85 |  | Il1b | Mus musculus | | NM_008361.3:108 | T005 | |  |
| TNF-a (Tumor necrosis factor alpha) | NM_013693.2 | 515-614 | TGGATCTCAAAGACAACCAACTAGTGGTGCCAGCCGATGGGTTGTACCTTGTCTACTCCCAGGTTCTCTTCAAGGGACAAGGCTGCCCCGACTACGTGCT | 85 | 85 |  | Tnf | Mus musculus | | NM_013693.2:514 | T006 | |  |
| IL-10 (Interleukin 10) | NM_010548.2 | 251-350 | GACAACATACTGCTAACCGACTCCTTAATGCAGGACTTTAAGGGTTACTTGGGTTGCCAAGCCTTATCGGAAATGATCCAGTTTTACCTGGTAGAAGTGA | 81 | 80 |  | Il10 | Mus musculus | | NM_010548.2:250 | T007 | |  |
| NPY (Neuropeptide Y) | NM_023456.2 | 231-330 | GACACTACATCAATCTCATCACCAGACAGAGATATGGCAAGAGATCCAGCCCTGAGACACTGATTTCAGACCTCTTAATGAAGGAAAGCACAGAAAACGC | 85 | 85 |  | Npy | Mus musculus | | NM_023456.2:230 | T008 | |  |
| NPY receptor Y1 | NM_010934.4 | 311-410 | TTCTCCACTTCTGGCTTTTGAAAATGATGACTGCCACCTGCCCTTGGCTGTGATATTCACCTTGGCTCTCGCTTATGGGGCGGTGATTATTCTTGGCGTC | 83 | 79 |  | Npy1r | Mus musculus | | NM_010934.4:310 | T009 | |  |
| NPY receptor Y2 | NM_001205099.1 | 591-690 | TCTACAGCCTTTCCACCCTGCTCATCCTGTACGTTTTGCCTCTGGGCATCATATCTTTCTCCTACACCCGTATCTGGAGTAAGCTGAGGAACCACGTCAG | 82 | 83 |  | Npy2r | Mus musculus | | NM_001205099.1:590 | T010 | |  |
| NPY receptor Y5 | NM_016708.3 | 725-824 | CCAGTGTTTCACAGCCTTGTGGAACTTAAGGAAACCTTTGGCTCAGCATTGCTAAGCAGCAAGTATTTGTGTGTTGAGTCATGGCCCTCTGATTCATACA | 80 | 79 |  | Npy5r | Mus musculus | | NM_016708.3:724 | T011 | |  |
| CRH (Corticotropin releasing hormone) | NM_205769.3 | 657-756 | CGCTGGAGAGGGAGAGGCGGTCGGAGGAGCCGCCCATCTCTCTGGATCTCACCTTCCACCTTCTGCGGGAAGTCTTGGAAATGGCCCGGGCAGAGCAGTT | 92 | 89 |  | Crh | Mus musculus | | NM_205769.3:656 | T012 | |  |
| BDNF (Brain derived neurotrophic factor) | NM_007540.4 | 640-739 | GTTCCACCAGGTGAGAAGAGTGATGACCATCCTTTTCCTTACTATGGTTATTTCATACTTCGGTTGCATGAAGGCGGCGCCCATGAAAGAAGTAAACGTC | 78 | 81 |  | Bdnf | Mus musculus | | NM_007540.4:639 | T013 | |  |
| TJP1 (Zonulin-1) | NM_009386.1 | 3566-3665 | GAGCAGCCGTCATACAGGTATGAGGTCTCAAGCTACACAGACCAGTTTTCTCGGAACTATGACCATCGCCTACGGTTTGAAGATCGAATCCCTACCTATG | 81 | 82 |  | Tjp1 | Mus musculus | | NM_009386.1:3565 | T014 | |  |
| OCLN (Occludin) | NM_008756.2 | 1421-1520 | CTGGGTCAGGGAATATCCACCTATCACTTCAGATCAACAAAGACAACTCTACAAGAGAAATTTTGATGCAGGTCTGCAGGAGTATAAGAGCTTACAGGCA | 84 | 83 |  | Ocln | Mus musculus | | NM_008756.2:1420 | T015 | |  |
| CLDN1 (Claudin-1) | NM_016674.4 | 411-510 | CTTCGACTCCTTGCTGAATCTGAACAGTACTTTGCAGGCAACCCGAGCCTTGATGGTAATTGGCATCCTGCTGGGGCTGATCGCAATCTTTGTGTCCACC | 82 | 82 |  | Cldn1 | Mus musculus | | NM_016674.4:410 | T016 | |  |
| CLDN5 (Claudin-5) | NM_013805.4 | 555-654 | CTCTGCTGGTTCGCCAACATCGTTGTCCGCGAGTTCTATGATCCGACGGTGCCGGTGTCACAGAAGTACGAGCTGGGCGCGGCGCTGTACATCGGCTGGG | 80 | 92 |  | Cldn5 | Mus musculus | | NM_013805.4:554 | T017 | |  |
| PPIL3 (Peptidyl-prolyl cis-trans isomerase-like 3 Reporter gene) | NM_027374.3 | 571-670 | GAGGAGGTAGCAGCATCTGGGCCAAAAAGTTTGAGGATGAATACAGTGAATATCTGAAGCACAATGTTCGAGGTGTTGTATCTATGGCTAATAATGGCCC | 83 | 80 | HK | Ppil3 | Mus musculus | | NM_027374.3:570 | T018 | |  |
| Ywhaz (Tyrosine 3-monooxygenase/tryptophan 5-monooxygenase activation protein zeta polypeptide) | NM_011740.2 | 456-555 | AACGTTGTAGGAGCCCGTAGGTCATCGTGGAGGGTCGTCTCAAGTATTGAGCAGAAGACGGAAGGTGCTGAGAAAAAGCAGCAGATGGCTCGAGAATACA | 80 | 82 | HK | Ywhaz | Mus musculus | | NM_011740.2:455 | T019 | |  |
| Tubb5 (Tubulin beta 5 class ) | NM_011655.5 | 407-506 | AGCCACAGGTGGCAAGTATGTCCCTCGAGCTATCTTGGTGGATCTAGAACCTGGGACTATGGACTCCGTTCGCTCAGGTCCTTTTGGCCAGATCTTCAGA | 85 | 83 | HK | Tubb5 | Mus musculus | | NM_011655.5:406 | T020 | |  |
| UBE2D2 (Ubiquitin-conjugating enzyme E2 D2 Reporter gene) | NM_019912.1 | 2245-2344 | TTCATGTGAACTAGGCTAGTTACCTCCCCTCGTCCCCTTTCCTAACCTAAATGTAAGCCAGGCCAGCTTGAAGGCCAATGCTCTTTAGTCACCAAGATCT | 80 | 83 | HK | Ube2d2a | Mus musculus | | NM_019912.1:2244 | T021 | |  |
| NR3C1 (Glucocorticoid receptor) | NM_008173.3 | 1801-1900 | ACCAGGATTCAGAAACTTACACCTGGATGACCAAATGACCCTTCTACAGTACTCATGGATGTTTCTCATGGCATTTGCCCTGGGTTGGAGATCATACAGA | 81 | 81 |  | Nr3c1 | Mus musculus | | NM_008173.3:1800 | T022 | |  |
| GRIN2B (Glutamate receptor NMDA2B) | NM_008171.3 | 1741-1840 | GAAGAGGATCTACCAGTCTAACATGCTGAATAGGTATCTGATCAACGTCACTTTTGAAGGGAGAAACCTGTCCTTCAGTGAAGATGGCTACCAGATGCAT | 82 | 82 |  | Grin2b | Mus musculus | | NM_008171.3:1740 | T023 | |  |
| CCL2 (CC-chemokine ligand 2) | NM_011333.3 | 416-515 | TCTTCAGCACCTTTGAATGTGAAGTTGACCCGTAAATCTGAAGCTAATGCATCCACTACCTTTTCCACAACCACCTCAAGCACTTCTGTAGGAGTGACCA | 79 | 82 |  | Ccl2 | Mus musculus | | NM_011333.3:415 | T024 | |  |
